# ASIC1a inhibits cell pyroptosis induced by acid‐induced activation of rat hepatic stellate cells

**DOI:** 10.1002/2211-5463.12850

**Published:** 2020-04-28

**Authors:** Jun Li, Lingjin Kong, Huiping Huang, Shaohua Luan, Rui Jin, Fanrong Wu

**Affiliations:** ^1^ School of Pharmacy Anhui Medical University Hefei China

**Keywords:** acid‐sensing ion channels, ASIC1a, hepatic fibrosis, hepatic stellate cells, pyroptosis

## Abstract

The activation of hepatic stellate cells (HSCs) is associated with liver fibrosis, the pathological feature of most forms of chronic hepatic damage, and is accompanied by abnormal deposition of the extracellular matrix (ECM). During the pathological process, acid‐sensing ion channel 1a (ASIC1a), which is responsible for Ca^2+^ transportation, is involved in the activation of HSCs. It has previously been identified that ASIC1a is related to pyroptosis in articular chondrocytes. However, it remains unclear whether ASIC1a restrains pyroptosis during liver fibrosis. Here, we determined that the levels of pyroptosis‐associated speck‐like protein, gasdermin D, caspase‐1, nucleotide‐binding oligomerization domain (NOD)‐like receptor 3, and apoptosis‐associated speck‐like protein (ASC) decreased, while the level of α‐smooth muscle actin and collagen‐I increased upon introduction of ASIC1a into an acid‐induced model. Inhibition or silencing of ASIC1a and the use of Ca^2+^‐free medium were able to promote the pyroptosis of activated HSCs, which reduced their deposition. In summary, our study indicates that ASIC1a inhibits pyroptosis of HSCs and that inhibition of ASIC1a may be able to promote pyroptosis to relieve liver fibrosis.

## Graphical abstract

Here, we found that acid‐sensing ion channel 1a (ASIC1a) inhibits the pyroptosis of acid‐induced activated hepatic stellate cells, which affects the degree of cell activation. Our results suggest that ASIC1a promotes Ca^2+^ influx and attenuates the assembly and expression of apoptosis‐associated speck‐like protein, nucleotide‐binding oligomerization domain (NOD)‐like receptor 3, and caspase‐1, thereby reducing the ability of gasdermin D to mediate pyroptosis.

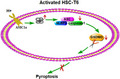

**Abbreviations**
ASC: apoptosis‐associated speck‐like proteins
ASICs: Acid‐sensitive ion channels
DMSO: dimethyl sulfoxide
ECM: extracellular matrix
GSDMD: gasdermin D
HSCs: hepatic stellate cells
IL‐1β/18: interleukin‐1β/18
LDH: lactate dehydrogenase
MTT: 3‐(4,5‐dimethylthiazol‐2‐yl)‐2,5‐diphenyltetrazolium bromide
NLRP3: nucleotide‐binding oligomerization domain (NOD)‐like receptor 3
PcTx‐1: psalmotoxin‐1
α‐SMA: α‐smooth muscle actin

Liver fibrosis is a chronic inflammatory disease caused by various pathogenic factors, and it is also a common pathological process of nonalcoholic fatty liver disease, alcoholic liver disease, and liver cancer. Activation of hepatic stellate cells (HSCs) is important in fibrotic progression, which involves a quiescent state change to myofibroblast‐like cells, accompanied by the appearance of α‐smooth muscle actin (α‐SMA) and excessive extracellular matrix (ECM) [1, 2]. However, there are currently no targeted drugs for hepatic stem cells, which limits current antifibrotic treatments [3].

Acid‐sensitive ion channels (ASICs) are extracellular acid‐activated cation channels related to the degenerin/epithelial Na^+^ channel family [4], which consists of seven members, acid‐sensing ion channel 1a (ASIC1a), 1b, 1b2, 2a, 2b, 3, and 4, encoded by four homologous genes. ASIC1a is a unique subunit that transports Ca^2+^, while the other ASICs only influence Na^+^ [5, 6, 7, 8]. Studies showed that ASICs could mediate some diseases, such as migraines associated with tumors, ischemic injury, inflammation, and so on. A common feature of these conditions is local tissue acidification and lower pH value [9]. Our previous studies showed that ASIC1a was expressed in rat liver and HSCs, and the level of ASIC1a expression was increased in acid‐activated HSCs, whereas blocking ASIC1a slowed the progression of liver fibrosis [10, 11].

Pyroptosis is an inflammatory form of programmed cell death [12]. It is characterized by the activation of caspase‐1, gasdermin D (GSDMD) [13, 14, 15], and the secretion of the proinflammatory cytokines interleukin (IL)‐1β and IL‐18 [16, 17, 18]. Recently, studies showed ASIC1a mediated pyroptosis in articular chondrocytes [19], in which nucleotide‐binding oligomerization domain (NOD)‐like receptor (NLR) family pyrin domain containing 3 (NLRP3) is activated and apoptosis‐associated speck‐like protein (ASC) forms a large spot‐like structure and recruits caspase‐1 protein, representing a classical signaling pathway of pyroptosis14 related to ATP, uric acid, double‐stranded DNA, Ca^2+^, bacterial toxins, and H^+^ [20, 21, 22, 23]. We hypothesized that ASIC1a could restrain the pyroptosis of activated HSCs; however, this required further verification.

In this study, we found that ASIC1a restrained the pyroptosis of activated HSCs, while the levels of NLRP3, ASC, caspase‐1, and GSDMD decreased. Furthermore, we investigated the effects of the inhibition and silencing of ASIC1a on activated HSCs, providing strong evidence for their influence. Moreover, we also demonstrated that Ca^2+^ plays a role in ASIC1a‐mediated pyroptosis.

## Material and methods

### Cell culture and cell treatment

Rat HSC line HSC‐T6 (Xiangya School of Medicine, Central South University) was cultured in Dulbecco’s modified Eagle’s medium supplemented with 10% FBS and 1% penicillin–streptomycin solution. HSCs were pre‐incubated with the ASIC1a‐specific inhibitor psalmotoxin‐1 (PcTx‐1; 100 nm; Sigma, St. Louis, MO, USA) for 1 h, or transfected with ASIC1a small‐interfering RNA (siRNA), ASIC1a expression plasmid, and Ca^2+^‐free medium (Thermo Fisher Scientific, Waltham, MA, USA) at pH 6.0 for 4 h. Then, the culture was changed to normal medium for 24 h. HSCs were grouped as follows: (i) control group (pH 7.4), model group (pH 6.0), pH 7.4 + PcTx‐1, pH 6.0 + PcTx‐1; (ii) control group (pH 7.4), model group (pH 6.0), pH 6.0 + control siRNA, pH 6.0 + ASIC1a siRNA; (iii) control group (pH 7.4), model group (pH 6.0), pH 6.0 + pcDNA3.1 NC, pH 6.0 + pcDNA3.1 ASIC1a; (iv) control group (pH 7.4), model group (pH 6.0), pH 6.0 + Ca^2+^‐free medium.

### Silencing and overexpression of ASIC1a in hepatic stellate cells

The HSCs were transfected with ASIC1a siRNA‐3 (Table 1) (Hanheng, Shanghai, China). A full‐length ASIC1a expression plasmid, which was transfected into pcDNA3.1 ASIC1a, was utilized to overexpress ASIC1a (Hanheng). HSC‐T6 was transiently transfected with the Lipofectamine 2000 (Thermo Fisher Scientific) and Opti‐MEM (Gibco, Thermo Fisher Scientific) reagents according to the manufacturer's protocol. After 24 h, cells were harvested and used for experiments.

**Table 1:** Primer sequences of ASIC1a small‐interfering oligos.

Gene	Forward primer	Reverse primer
ASIC1a‐si‐1	5 ՛ ‐GGACAUUCAGCAAGAUGAA‐3 ՛	5 ՛ ‐UUCAUCUUGCUGAAUGUCC ‐3 ՛
ASIC1a‐si‐2	5 ՛ ‐GGUCAAGAUCCCAAGCAAA ‐3 ՛	5 ՛ ‐UUUGCUUGGGAUCUUGACC ‐3 ՛
ASIC1a‐si‐3	5 ՛ ‐GCGUGAAUUCUACGACAGA ‐3 ՛	5 ՛ ‐UCUGUCGUAGAAUUCACGC ‐3 ՛

### Real‐time quantitative PCR (qPCR)

Trizol reagent (Invitrogen, Carlsbad, CA, USA) was used to collect total RNA of HSC‐T6 with chloroform. One microgram of total mRNA was used for reverse transcription of cDNA according to the manufacturer’s instructions (TAKARA, Dalian, China). The cDNA analysis was performed on a PikoReal 96 qPCR system (Thermo Fisher Scientific) using SYBR Premix Ex Taq II (TAKARA). The cDNA system was firstly denatured at 94 °C for 5 min, held at 94 °C for 40 s, annealed at 54, 52, 53, and 51 °C for 40 s, extended at 72 °C for 1 min for 35 cycles, and then kept at 72 °C for 30 s. The primer sequences used (Shanghai Biology Engineering Corporation, Songjiang district, Shanghai, China) are shown in Table 2. After 30 s at 72 °C, the expression of mRNA was calculated using the $2^{-\Delta \Delta C_{t}}$ method.

**Table 2:** Primer sequences of several genes for real‐time PCR.

Gene	Forward primer	Reverse primer
β‐actin	5 ՛ ‐GAGCGCAAGTACTCTGTGTG‐3 ՛	5 ՛ ‐CCTGCTTGCTGATCCACATC‐3 ՛
ASIC1a	5 ՛ ‐CGGCTGAAGACCATGAAAGG‐3 ՛	5 ՛ ‐AAGGATGTCTCGTCGGTCTC‐3 ՛
α‐SMA	5 ՛ ‐GAGGGATCCTGACCCTGAAG‐3 ՛	5 ՛ ‐CCACGCGAAGCTCGTTATAG‐3 ՛
Collagen‐I	5 ՛ ‐ACCTCAGGGTATTGCTGGAC‐3 ՛	5 ՛ ‐GACCAGGGAAGCCTCTTTCT‐3 ՛
GSDMD	5 ՛ ‐TGCGGGAGTGGTCAAGAA‐3 ՛	5 ՛ ‐TGCTCAGGAGGCAGTAGGG‐3 ՛
Caspase‐1	5 ՛ ‐TGCCTGGTCTTGCGACTTGGA‐3 ՛	5 ՛ ‐ATGTCCTGGGAAGAGGTAGAAACG‐3 ՛
ASC	5 ՛ ‐CACAGCCAGAACAGAACAT‐3 ՛	5 ՛ ‐TCAGCACATTGCCATACA‐3 ՛
NLRP3	5 ՛ ‐CCCAGGCTGTAACATTCA‐3 ՛	5 ՛ ‐GCTTCAGTCCCACGCACA‐3 ՛

### Western blotting

The treated HSCs were lysed by adding 500 μL of radioimmunoprecipitation assay (Beyotime Institute of Biotechnology, Beyotime Biotechnology, Shanghai, China) buffer and 5 μL of phenylmethylsulfonyl fluoride (Beyotime Biotechnology) on ice for 30 min and then centrifuged at 12 000 ***g*** for 30 min at 4 °C. The supernatant was removed, and the protein was quantified using a bicinchoninic acid protein assay kit (Beyotime, Biotechnology). After polyacrylamide gel electrophoresis, the protein was transferred to a polyvinylidene fluoride (Millipore, Billerica, MA, USA) membrane by electroporation. The membrane was incubated with 5% milk for 3 h and then probed with specific primary affinity‐purified antibodies against β‐actin (rabbit, 1 : 1000; Affinity Biocsience, Cincinnati, OH, USA), ASIC1a (rabbit, 1 : 1000; Abcam, Cambridge, MA, USA), collagen‐I (mouse, 1 : 1000, Bioss, Wuhan, China), α‐SMA (mouse, 1 : 1000, Bioss), caspase‐1 (rabbit, 1 : 500; Abcam), ASC (rabbit, 1 : 1000, Abcam), NLRP3 (rabbit, 1 : 1000; Abcam), and GSDMD (rabbit, 1 : 1000; Abcam) overnight at 4 °C. After washing three times in Tris‐buffered saline(TBS)/Tween‐20, the membrane was incubated with horseradish peroxidase‐conjugated rabbit anti‐mouse (1 : 10 000; OriGene Technologies, Beijing, China) or goat anti‐rabbit immunoglobulin G (IgG; 1 : 10 000; OriGene Technologies) at 37 °C for 1 h. Membranes were also washed with TBS/Tween‐20 three times and then detected using an enhanced chemiluminescence kit (Thermo Fisher Scientific). Using β‐actin as an internal reference, the relative expression levels of the proteins were expressed as the ratio of the corresponding protein to β‐actin using imaging analysis software (Media Cybernetics, Rockville, MD, USA).

### Enzyme‐linked immunosorbent assay (ELISA) of IL‐1β and IL‐18

The medium of each treatment group was collected and centrifuged at 1000 ***g*** for 20 min at 4 °C to remove impurities and cell debris. According to the instructions of the rat IL‐1β and IL‐18 detection kit (Elabscience, Wuhan, China), an ELISA was performed. The absorbance of the microplate reader was set to 450 nm, and the standard curve was generated using a standard product to determine the concentration of each culture of IL‐1β and IL‐18 in the supernatant.

### Lactate dehydrogenase (LDH) release assay

The LDH assay was performed according to the LDH Measurement Kit Instructions (Beyotime Biotechnology). The cells were seeded on 96‐well plates for the logarithmic growth phase and divided into a negative control group, maximum active well, and PcTx‐1 treatment group. The LDH release reagent was added 1 h before the scheduled time, and incubation was continued in the incubator. The 96‐well plate was centrifuged at 400 ***g*** for 5 min, and then, 120 µL of the supernatant was aspirated into a new 96‐well plate, before measuring the absorbance at a wavelength of 490 nm using a microplate reader to calculate the LDH content.

### MTT assay

Hepatic stellate cells was seeded at a density of 5 × 10^3^ cells per mL in 96‐well plates. After the cells returned to normal morphology, they were treated with PcTx‐1 for 1 h, stimulated with pH 6.0 medium for 4 h, and then replaced with normal culture. The cells were cultured for 24 h, and after add in MTT(Beyotime Biotechnology) 4h, add in 150 uL DMSO solution, the survival rate was measured at a wavelength of 490 nm using a microplate reader.

### Laser scanning confocal microscopy for intracellular [Ca^2+^]_i_

The cells were incubated with 3 mm Fluo‐3‐am (Dojindo Laboratories, Mashikimachi, Japan) and 0.04% Pluronic F‐127 (Biotium, Fremont, CA, USA) for 30 min at 37 °C, and then washed with fluorescent dye, before adding D‐Hanks solution to equilibrate the cells for 10 min. The intensity of intracellular Ca^2+^ fluorescence was measured by laser confocal microscopy (Carl‐Zeiss, Jena, Germany). The acidic Hanks solution (pH 6.0) was directly added using a pipette. The recording continued throughout the continued addition of the acidic Hanks solution and lasted for approximately 5 min. The intensity of the fluorescence of individual cells was measured using the lsm 5 image software(Carl‐Zeiss).

### Statistical analysis

The experimental data were evaluated by calculating the mean ± SD. Statistical analyses were performed using spss 16.0 software (SPSS Inc., Chicago, IL, USA). One‐way ANOVA was used to assess differences between multiple groups. Differences between the two groups were compared using Student's *t*‐test. The significance level was set to *P* < 0.05.

## Results

### ASIC1a promotes hepatic stellate cells activation

**Fig 1:**
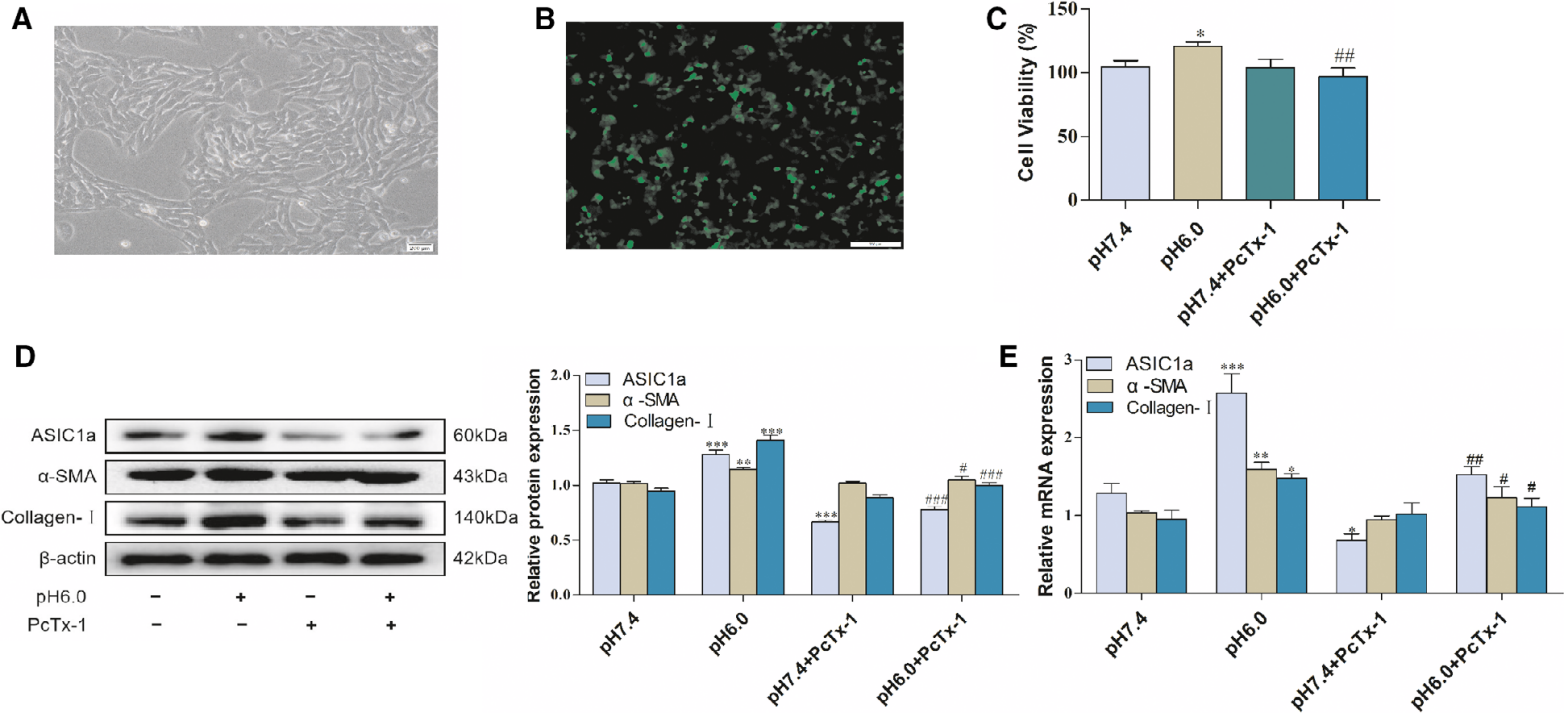
HSCs, transfection efficiency, and effect of PcTx‐1 on viability in HSCs: (A) morphology of hepatic stellate (scale bar, 200 μm); (B) transfection ASIC1a siRNA (scale bar, 200 μm); (C) MTT analysis of cell viability of HSCs under PcTx‐1; (D) western blotting analysis and densitometric quantification of ASIC1a, α‐SMA, and collagen‐I protein levels in HSCs under PcTx‐1; (E) mRNA levels of ASIC1a, α‐SMA, and collagen‐I in HSCs under PcTx‐1. Satistical analyses were performed using Student's *t*‐test. Data are expressed as the mean ± SEM (*n* = 4). ^*^ *P* < 0.05, ^**^ *P* < 0.01, ^***^ *P* < 0.001 vs. pH 7.4 group; ^#^ *P* < 0.05, ^##^ *P* < 0.01, ^###^ *P* < 0.001vs. pH 6.0 group.

**Fig 2:**
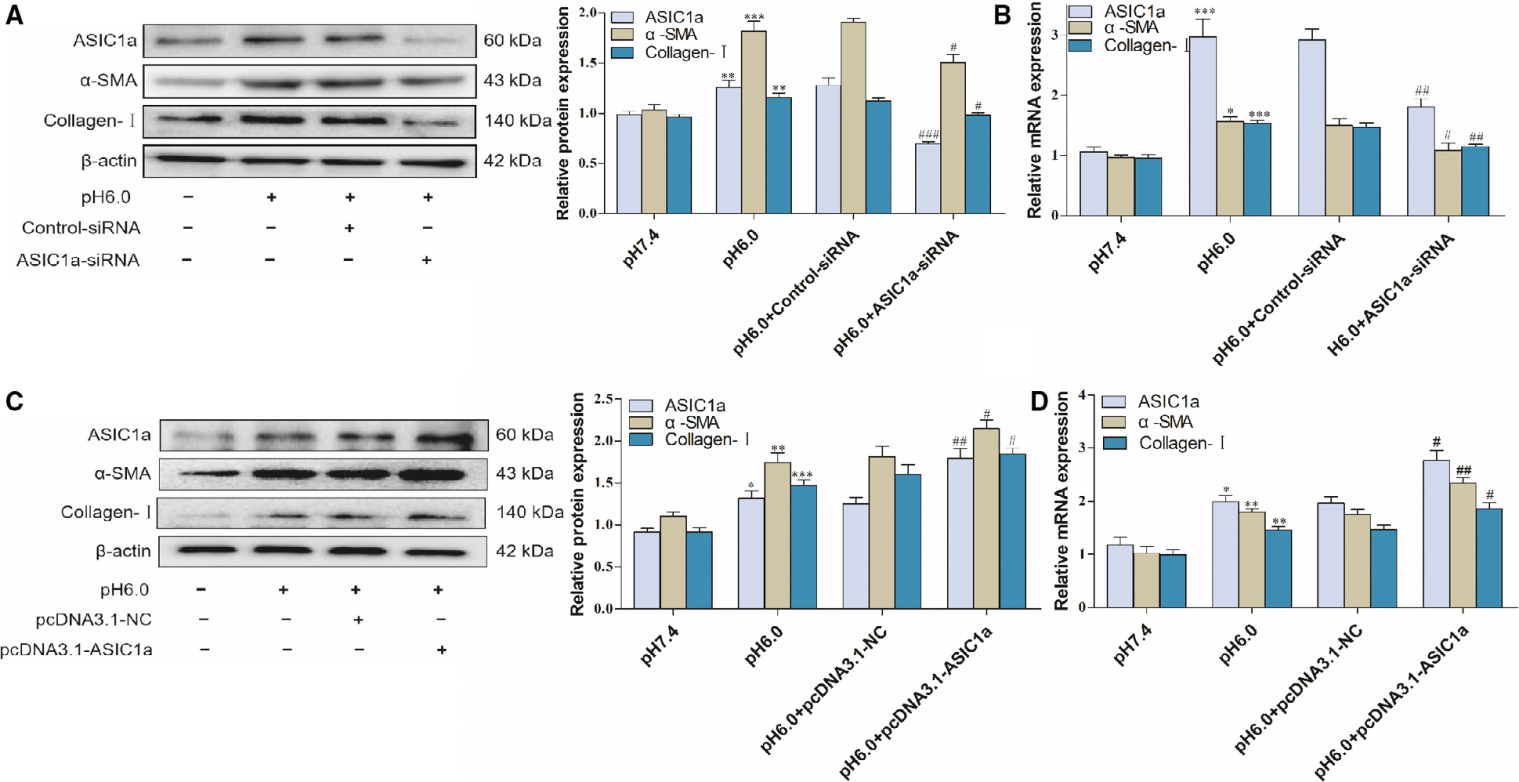
Effect of ASIC1a siRNA and ASIC1a expression plasmid on viability in HSCs: (A) western blotting analysis and densitometric quantification of ASIC1a, α‐SMA, and collagen‐I protein levels in HSCs after transfecting ASIC1a siRNA; (B) mRNA levels of ASIC1a, α‐SMA, and collagen‐I in HSCs after transfecting ASIC1a siRNA; (C) western blotting analysis densitometric quantification of ASIC1a, α‐SMA, and collagen‐I protein levels in HSCs after transfecting ASIC1a expression plasmid; (D) mRNA levels of ASIC1a, α‐SMA, and collagen‐I in HSCs after transfecting ASIC1a expression plasmid. Satistical analyses were performed using Student's *t*‐test. Data are expressed as the mean ± SEM (*n* = 4). ^*^ *P* < 0.05, ^**^ *P* < 0.01, ^***^ *P* < 0.001 vs. pH 7.4 group; ^#^ *P* < 0.05, ^##^ *P* < 0.01, ^###^ *P* < 0.001 vs. pH 6.0 group.

We transfected fluorescent ASIC1a siRNA into rat HSCs (Fig. 1A,B). The effect of extracellular acids on the viability of HSCs was measured using the 3‐(4,5‐dimethylthiazol‐2‐yl)‐2,5‐diphenyltetrazolium bromide (MTT) method, and the results show that cell viability increased under acidification (Fig. 1C). Our previous study found that ASIC1a expression is increased in acid‐induced HSCs. The results also show that acidification of HSC‐T6 to pH 6.0 could open ASIC1a channels and increase their mRNA and protein expression. In addition, western blotting and real‐time PCR analysis showed that α‐SMA and collagen‐I protein and mRNA were significantly increased under acid stimulation (Figs 1D,E and 2A,B). This analysis also indicated that α‐SMA, collagen‐I of PcTx‐1 (an inhibitor of ASIC1a), and ASIC1a siRNA groups could be suppressed, thereby reducing the activity of HSCs. The results of the ASIC1a expression plasmid group were opposite to the above (Fig 2C,D). These data show that acidic stimulation of cells could open ASIC1a channels and promote the level of HSC activation.

### Extracellular acid activates ASIC1a to promote Ca^2+^ influx

**Fig 3:**
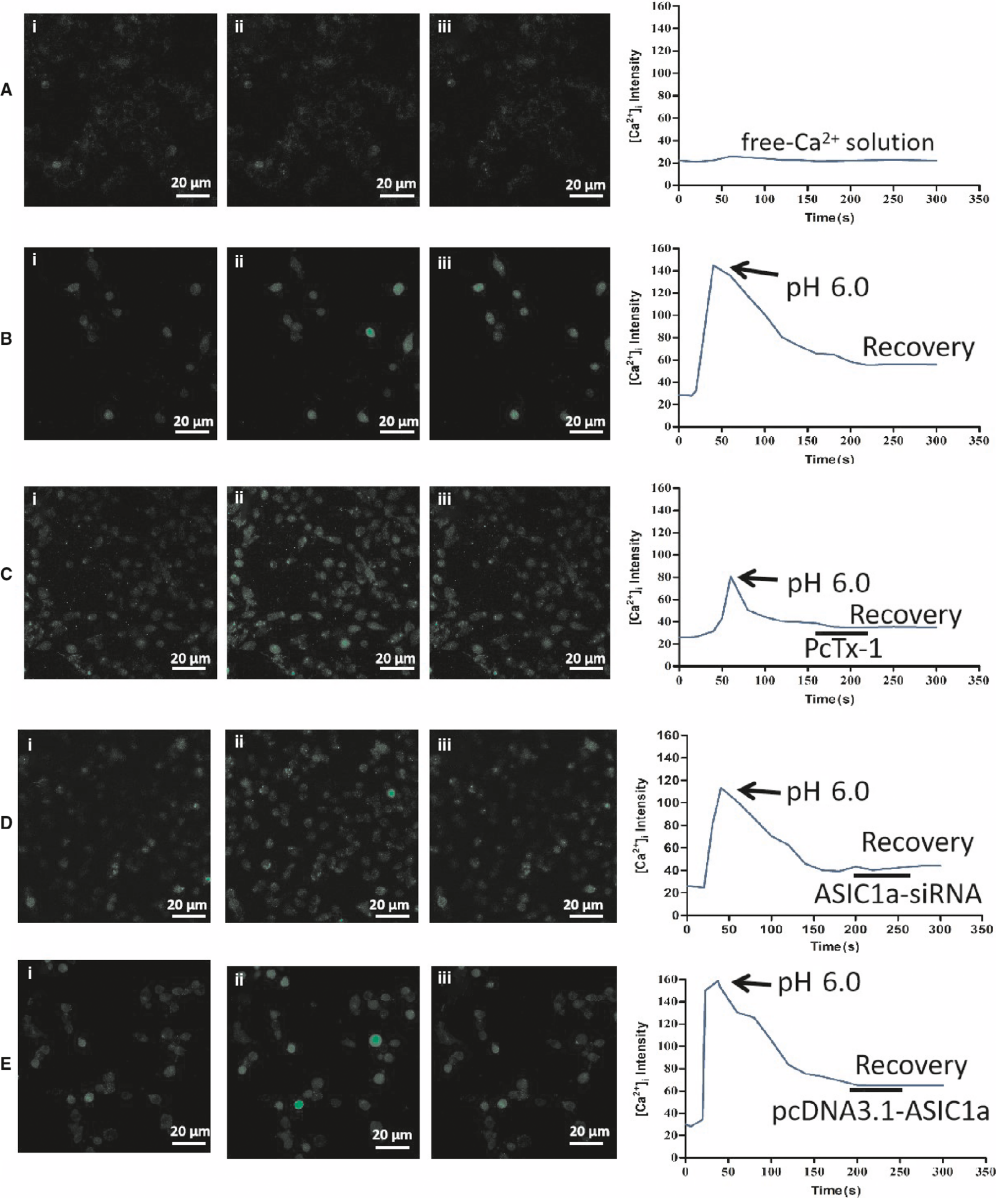
Effect of ASIC1a on Ca^2+^ influx: (A) acid‐induced elevation of [Ca^2+^]_i_ in Ca^2+^‐free extracellular solution (scale bar, 20 μm); (B) acid‐induced elevation of [Ca^2+^]_i_ in extracellular Ca^2+^ solution (scale bar, 20 μm); (C) acid‐induced elevation of [Ca^2+^]_i_ in HSCs treated with PcTx‐1 (scale bar, 20 μm); (D) acid‐induced elevation of [Ca^2+^]_i_ in HSCs treated with ASIC1a siRNA (scale bar, 20 μm); (E) acid‐induced elevation of [Ca^2+^]_i_ in HSCs treated with ASIC1a expression plasmid (scale bar, 20 μm).

We investigated the changes in [Ca^2+^]_i_ in rat HSCs treated with extracellular acidosis. Based on confocal laser scanning microscopy, our results show that the application of an extracellular solution acidulated to pH 6.0 significantly increased [Ca^2+^]_i_ in rat HSCs compared with the normal group (Fig. 3A,B). The ASIC1a‐specific inhibitor PcTx‐1 and ASIC1a siRNA reduced the amplitude of the [Ca^2+^]_i_ response to extracellular acid (Fig 3C,D), whereas the results of the ASIC1a expression plasmid group were opposite to the above (Fig. 3E). Therefore, the results indicate that Ca^2+^ is obviously associated with ASIC1a.

### ASIC1a‐mediated Ca^2+^ inhibits the pyroptosis of activated hepatic stellate cells

**Fig 4:**
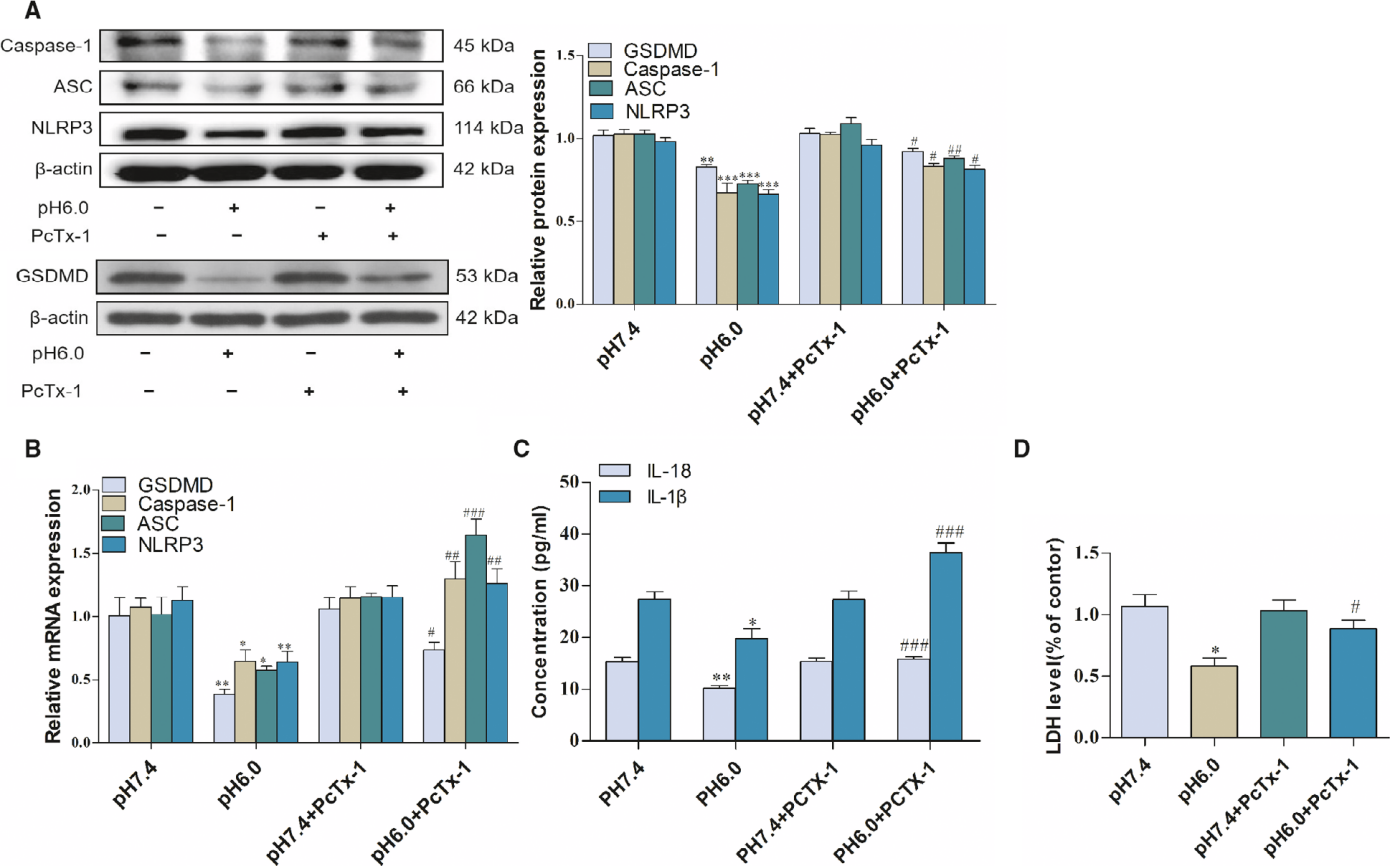
Effect of PcTx‐1 on the pyroptosis of acid‐induced activated HSCs: (A) Western blotting analysis and densitometric quantification of caspase‐1, ASC, NLRP3, and GSDMD protein levels in HSCs under PcTx‐1; (B) mRNA levels of caspase‐1, ASC, NLRP3, and GSDMD in HSCs under PcTx‐1; (C) IL‐1β and IL‐18 levels of cell supernatant under PcTx‐1; (D) LDH levels of cell supernatant under PcTx‐1. Satistical analyses were performed using Student's *t*‐test. Data are expressed as the mean ± SEM (*n* = 4).^*^ *P* < 0.05, ^**^ *P* < 0.01, ^***^ *P* < 0.001 vs pH 7.4 group; ^#^ *P* < 0.05, ^##^ *P* < 0.01, ^###^ *P* < 0.001 vs. pH 6.0 group.

**Fig 5:**
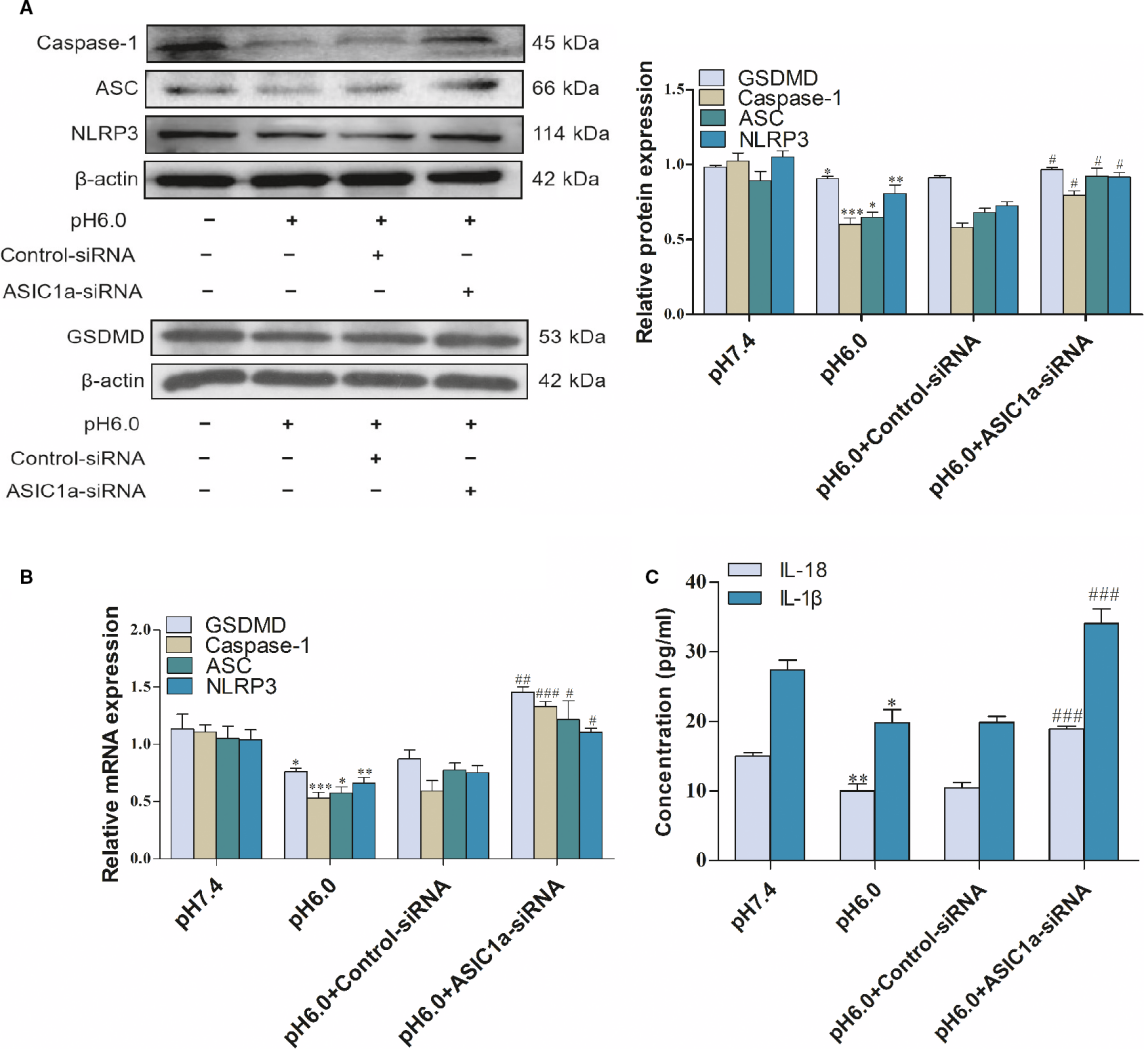
Effect of ASIC1a siRNA on the pyroptosis of acid‐induced activated HSCs: (A) western blotting analysis and densitometric quantification of caspase‐1, ASC, NLRP3, and GSDMD protein levels in HSCs after transfecting ASIC1a siRNA; (B) mRNA levels of caspase‐1, ASC, NLRP3, and GSDMD in HSCs after transfecting ASIC1a siRNA; (C) IL‐1β and IL‐18 levels of cell supernatant after transfecting ASIC1a siRNA. Statistical analyses were performed using Student's *t*‐test. Data are expressed as the mean ± SEM (*n* = 4). *P < 0.05, **P < 0.01, ^***^ *P* < 0.01 vs. pH 7.4 group; ^#^ *P* < 0.05, ^##^ *P* < 0.01, ^###^ *P* < 0.001 vs. pH 6.0 group.

**Fig 6:**
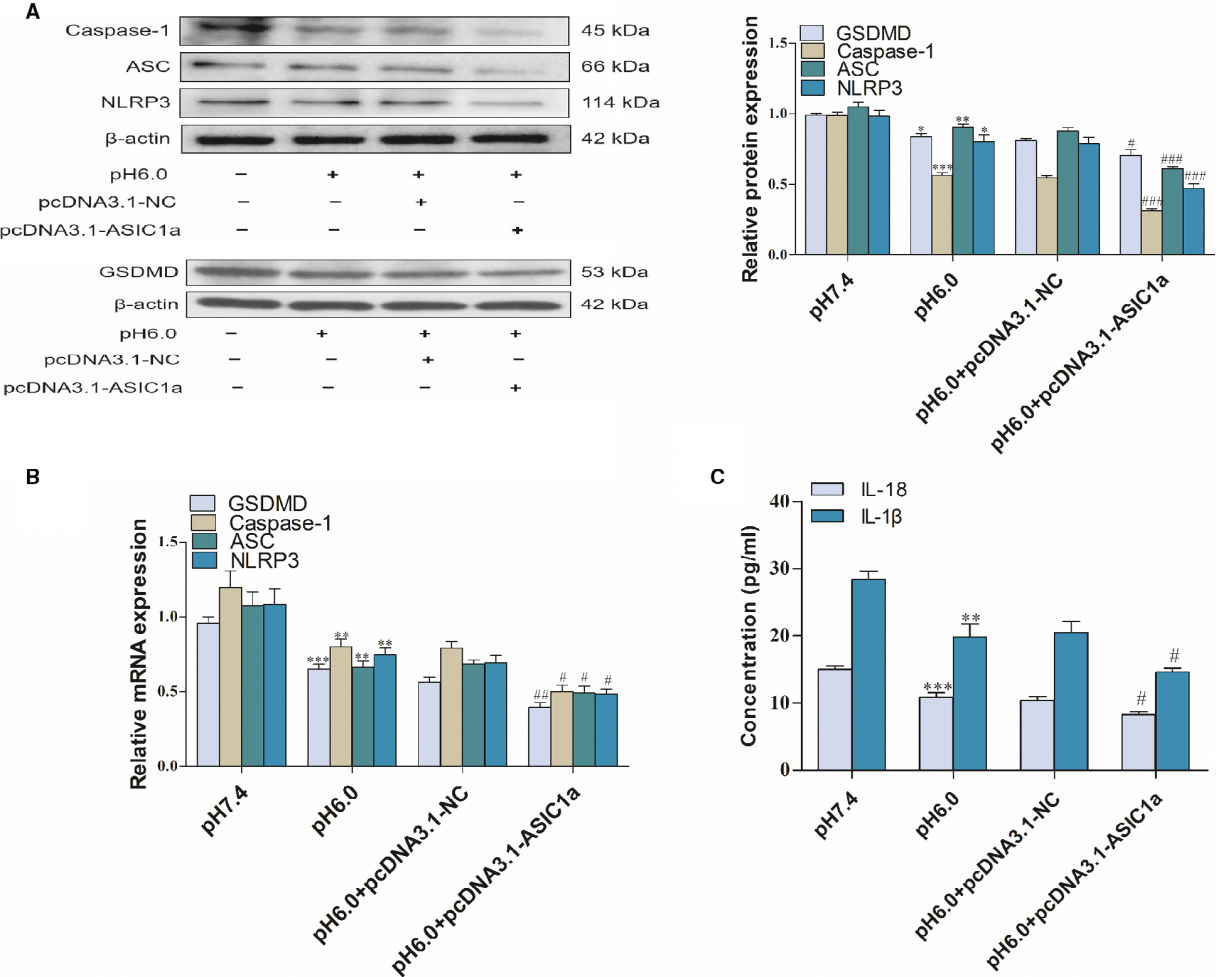
Effect of ASIC1a expression plasmid on the pyroptosis of acid‐induced activated HSCs: (A) western blotting analysis and densitometric quantification of caspase‐1, ASC, NLRP3, and GSDMD protein levels in HSCs after transfecting ASIC1a expression plasmid; (B) mRNA levels of caspase‐1, ASC, NLRP3, and GSDMD in HSCs after transfecting ASIC1a expression plasmid; (C) IL‐1β and IL‐18 levels of cell supernatant after transfecting ASIC1a expression plasmid. Statistical analyses were performed using Student's *t*‐test. Data are expressed as the mean ± SEM (*n* = 4). *P < 0.05, **P < 0.01, ^***^ *P* < 0.001 vs. pH 7.4 group; ^#^ *P* < 0.05, ^##^ *P* < 0.01, ^###^ *P* < 0.001 vs. pH 6.0 group.

As mentioned previously, studies showed that ASIC1a is closely related to pyroptosis articular chondrocytes. Therefore, we investigated the effects of ASIC1a on the pyroptosis of activated HSCs. Upon introducing ASIC1a in the acid‐induced model group, the mRNA and protein expression levels of caspase‐1, ASC, NLRP3, and GSDMD were decreased compared with the control group. The study also indicates that the ASIC1a‐specific inhibitor PcTx‐1 group and the ASIC1a siRNA group enhanced the level of pyroptosis compared to the model group through the detection of caspase‐1, ASC, NLRP3, and GSDMD in terms of mRNA and protein expression (Figs 4A,B, and 5A,B). ELISA results of the expression of IL‐1β and IL‐18 in the culture medium indicate that, compared with the control group, IL‐1β and IL‐18 in the model group were significantly reduced, while, in the PcTx‐1 group and ASIC1a siRNA group, they could effectively inhibit the reduction (Figs 4C and 5C). In addition, as an indicator of pyroptosis, our results also show that acidosis could reduce the release of LDH at pH 6.0, whereas the PcTx‐1 group increased its release compared with the pH 6.0 group (Fig. 4D). Interestingly, in terms of caspase‐1, ASC, NLRP3, GSDMD, IL‐1β, and IL‐18 pyroptosis‐related indicators, we can see that the ASIC1a expression plasmid group continued to reduce the level of pyroptosis compared with the model group (Fig. 6A–C). These results indicate that ASIC1a is involved in the pyroptosis of activated HSCs, and the inhibition of ASIC1a can promote the pyroptosis of activated HSCs.

**Fig 7:**
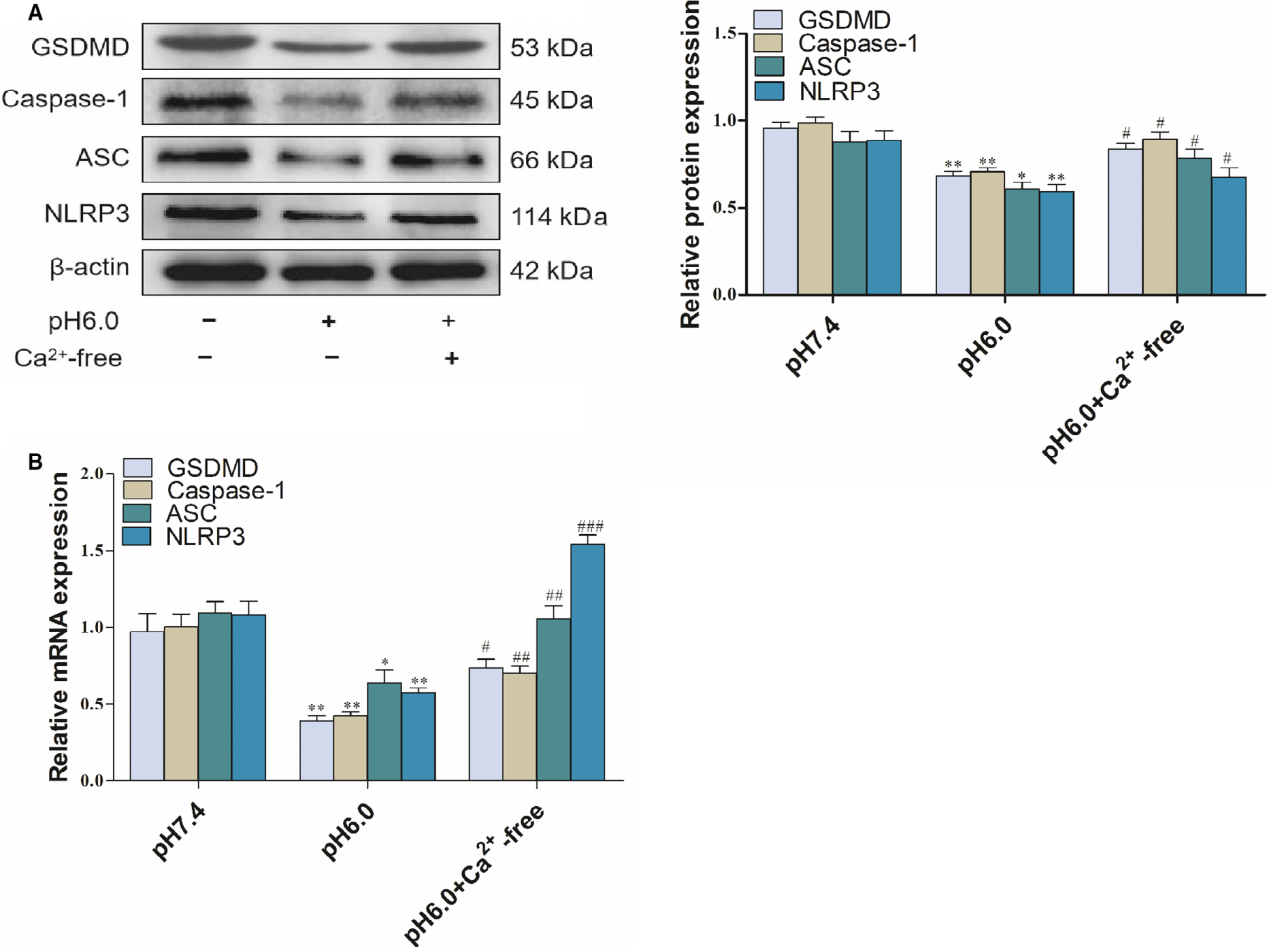
Role of Ca^2+^ in pyroptosis in activated HSCs: (A) western blotting analysis and densitometric quantification of GSDMD, caspase‐1, ASC and NLRP3, protein levels in HSCs in Ca^2+^‐free medium; (B) mRNA levels of NLRP3 in HSCs in Ca^2+^‐free medium. Statistical analyses were performed using Student's *t*‐test. Data are expressed as the mean ± SD (*n* = 4). *P < 0.05, **P < 0.01 vs. pH 7.4 group; ^#^ *P* < 0.05, ^##^ *P* < 0.01 vs. pH 6.0 group.

As previously mentioned, the concentration of intracellular Ca^2+^ is closely associated with ASIC1a in HSCs. We investigated whether Ca^2+^ concentration is important for ASIC1a‐mediated pyroptosis in activated HSCs. Results indicated that pretreatment with Ca^2+^‐free medium could enhance the pyroptosis of activated HSCs based on the expression of caspase‐1, ASC, NLRP3, and GSDMD compared with the pH 6.0 group (Fig. 7A), in accordance with the PCR results (Fig. 7B). As a result, we conclude that ASIC1a restrains the pyroptosis of activated HSCs by regulating [Ca^2+^]_i_.

## Discussions

Liver fibrosis, the pathological feature of most forms of chronic hepatic damage, is responsible for much morbidity and mortality worldwide [24, 25]. The occurrence and development of liver fibrosis is a multipath and multifactor participation process. HSCs play an important role in the development of liver fibrosis [24, 26]. The main pathological change involves excessive synthesis and abnormal deposition of the ECM. Therefore, the accumulation of activated HSCs can be inhibited by modulating their stimulation or proliferation or by promoting their death, and they represent the leading target in preventing liver fibrosis [27].

Acid‐sensing ion channel 1a is a proton‐gated ion channel for Ca^2+^ transportation. It is expressed in the mammalian nervous system and other tissues, in which it exerts pathophysiological effects [28]. Our previous studies showed that ASIC1a is highly expressed in hepatic fibrosis rat tissues and platelet‐derived growth factor (PDGF)–BB‐induced HSCs, and ASIC1a channels promote liver fibrosis by increasing intracellular Ca^2+^ [10, 11]. In our study, RT‐PCR and western blotting data also showed that, under acid stimulation, the ASIC1a channel was open, and HSC‐T6 was activated, thus suggesting an increase in the expression of α‐SMA and collagen‐I. These results could be prevented by PcTx‐1 and transfected ASIC1a siRNA. Moreover, the MTT result showed that the activity of HSCs was increased under acidic conditions. A previous study indicated that collagen deposition mainly accounts for proteins in fibrotic liver [29]; thus, we can say that the inhibition of ASIC1a may relieve acid‐induced activated HSC activity. Our results also showed that the ASIC1a expression plasmid group aggravated the acid‐induced cell activation. This phenomenon was consistent with our previous studies, showing that ASIC1a exerts a key role in liver fibrosis.

In the regulation of cell function, Ca^2+^ plays a key role as a second messenger. At the same time, Ca^2+^ is also one of a variety of activation molecules involved with proteins, phospholipids, and nucleases. Current studies found that Ca^2+^ and cell proliferation, differentiation, movement, secretion, and apoptosis are related [30]. In liver fibrosis, the Ca^2+^ signal plays a key role in the activation of HSCs [31]. In our study, it was revealed by laser confocal results that ASIC1a mediates the permeability of Ca^2+^. Under acid stimulation, the ASIC1a‐gated channel was open, and Ca^2+^ influx was increased compared with the normal group. Moreover, the inhibition and silencing of ASIC1a reduced the flow of Ca^2+^, while the ASIC1a expression plasmid group had the opposite result. In this study, we demonstrated that Ca^2+^ and ASIC1a are closely related, and they are also both involved in the ASIC1a‐mediated acid activation of HSCs.

Recent studies showed that pyroptosis is closely related to some liver‐related diseases such as liver fibrosis, liver cancer, and alcohol hepatitis [32, 33, 34]. Previously, pyroptosis was shown to be associated with liver fibrosis in nonalcoholic steatohepatitis [35]. Another report indicated that the activation of inflammatory bodies can lead to the death of hepatocytes, thereby further inducing liver inflammation and liver fibrosis [32]. There are a small number of inflammatory bodies in HSCs, and the inhibition of the formation of inflammatory bodies in HSCs can downregulate the content of collagen and α‐SMA, as well as the formation and deposition of the ECM, which are important for liver fibrosis [36]. However, further research is needed on whether or not their inhibition can result in their death. GSDMD and GSDME are well‐defined pyroptosis performers, while caspase‐4 caspase‐1, caspase‐5, and caspase‐11 can activate GSDMD [37, 38, 39]. However, GSDMDE is only activated by caspase‐3 [40, 41, 42]. Caspase‐1‐dependent GSDMD cleavage is defined as the classical pyroptosis pathway [39]. Research showed that pyroptosis is associated with dependence on the caspase‐1 pathway in the liver [43, 44]. In our study, the decreased level of pyroptosis under acidic conditions could be determined from the decrease in mRNA and protein levels of caspase‐1, ASC, NLRP3, and GSDMD, accompanied by an increase in the levels of collagen‐I and α‐SMA. The release of LDH was also downregulated. Furthermore, as indicators of pyroptosis, IL‐1β and IL‐18 levels in the culture medium also decreased in the model group compared with the control group. Thus, we can infer that that pyroptosis is related to activated HSCs; however, the above results with regard to pyroptosis could be negated through the addition of PcTx‐1 and transfected ASIC1a siRNA, accompanied by an attenuation of HSC activation. Therefore, ASIC1a participated in the pyroptosis of acid‐induced activated HSCs. In brief, it can be said that the inhibition of ASIC1a in this study showed a great effect in terms of increasing pyroptosis and reducing the degree of activation of HSCs, and the ASIC1a expression plasmid group result also provided further evidence for our conclusion.

As mentioned above, with the change in pyroptosis‐related indicators, the expression of inflammatory corpuscle components (NLRP3, ASC, caspase‐1) also changed, and we previously demonstrated that Ca^2+^ and ASIC1a are closely related. Thus, we then investigated whether increasing intracellular [Ca^2+^]_i_ mediated by ASIC1a is associated with the pyroptosis of activated HSCs. In this study, we found that the Ca^2+^‐free medium could promote GSDMD, caspase‐1, ASC, and NLRP3 levels in activated HSCs compared with the model group. It can be said Ca^2+^ is associated with the pyroptosis of activated HSCs; thus, we think that ASIC1a is involved in the pyroptosis of activated HSCs though inflammatory corpuscle components (NLRP3, ASC, caspase‐1), while their dependence on Ca^2+^ also played an important role.

**Fig 8:**
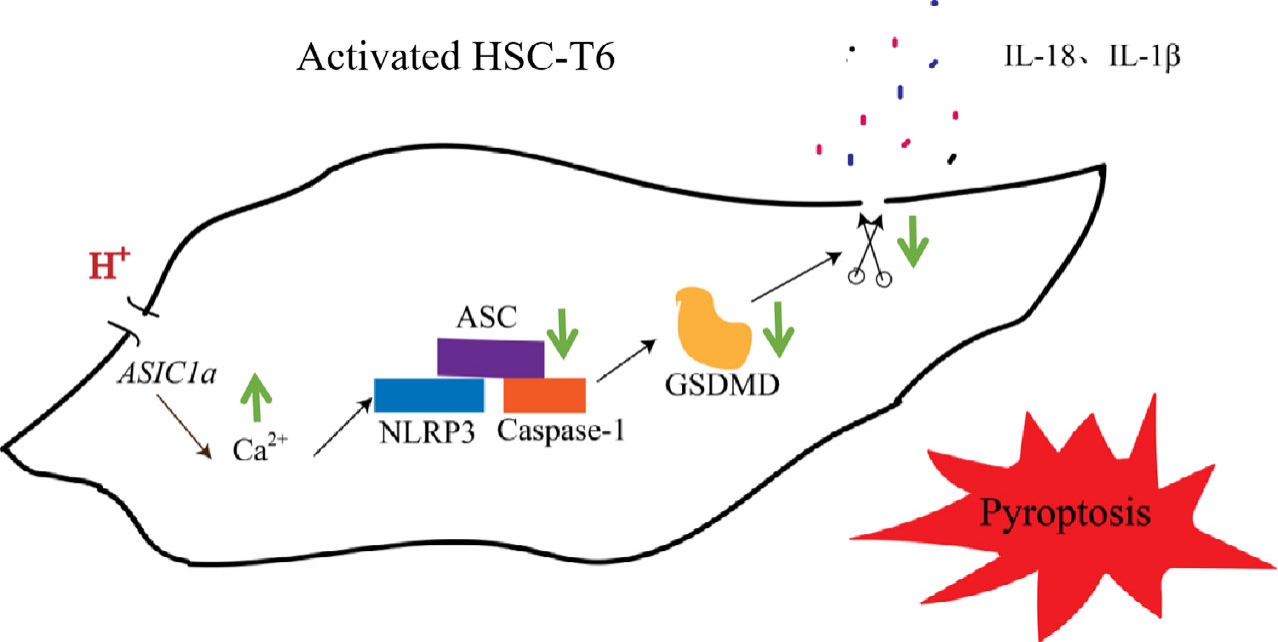
Schematic diagram of the potential mechanism of ASIC1a involved in the pyroptosis of activated HSCs. Small green arrows indicate enhancement (up arrow) or reduction (down arrow) in the schematic diagram.

In summary, our study demonstrated that rat hepatic stellate extracellular acidification activates ASIC1a and promotes Ca^2+^ influx into cells. This situation restrains the expression, polymerization, and assembly of the NLRP3 inflammasome, thereby reducing the pyroptosis of activated HSCs (Fig. 8). Although Ca^2+^ is associated with ASIC1a‐mediated pyroptosis in activated HSCs, other specific mechanisms may be involved in the process. Nonetheless, we conclude that it may be possible to develop effective drugs for liver fibrosis that target an improvement in the pyroptosis of activated HSCs.

## Conflict of interest

The authors declare no conflict of interest.

## Author contributions

JL performed the experiments and wrote the paper. L‐JK performed the experiments. H‐PH, S‐HL, and RJ analyzed the data. F‐RW designed the research and provided funding.

## Data Availability

All data generated or analyzed during this study are included in this published article.
